# Regulation of Liver Enriched Transcription Factors in Rat Hepatocytes Cultures on Collagen and EHS Sarcoma Matrices

**DOI:** 10.1371/journal.pone.0124867

**Published:** 2015-04-22

**Authors:** Jürgen Borlak, Prafull Kumar Singh, Ina Rittelmeyer

**Affiliations:** Centre for Pharmacology and Toxicology, Hannover Medical School, Hannover, Germany; Bambino Gesu' Children Hospital, ITALY

## Abstract

Liver-enriched transcription factors (LETF) play a crucial role in the control of liver-specific gene expression and for hepatocytes to retain their molecular and cellular functions complex interactions with extra cellular matrix (ECM) are required However, during cell isolation ECM interactions are disrupted and for hepatocytes to regain metabolic competency cells are cultured on ECM substrata. The regulation of LETFs in hepatocytes cultured on different ECM has not been studied in detail. We therefore compared two common sources of ECM and evaluated cellular morphology and hepatocyte differentiation by investigating DNA binding activity of LETFs at gene specific promoters and marker genes of hepatic metabolism. Furthermore, we studied testosterone metabolism and albumin synthesis to assess the metabolic competence of cell cultures. Despite significant difference in morphological appearance and except for HNF1β (p<0.001) most LETFs and several of their target genes did not differ in transcript expression after Bonferroni adjustment when cultured on collagen or Matrigel. Nonetheless, Western blotting revealed HNF1β, HNF3α, HNF3γ, HNF4α, HNF6 and the smaller subunits of C/EBPα and C/EBPβ to be more abundant on Matrigel cultured cells. Likewise, DNA binding activity of HNF3α, HNF3β, HNF4α, HNF6 and gene expression of hepatic lineage markers were increased on Matrigel cultured hepatocytes. To further investigate hepatic gene regulation, the effects of Aroclor 1254 treatment, e.g. a potent inducer of xenobiotic defense were studied *in vivo* and *in vitro*. The gene expression of C/EBP-α increased in rat liver and hepatocytes cultured on collagen and this treatment induced DNA binding activity of HNF4α, C/EBPα and C/EBPβ and gene expression of CYP1A1 and CYP1A2 *in vivo* and *in vitro*. Taken collectively, two sources of ECM greatly affected hepatocyte morphology, activity of liver enriched transcription factors, hepatic gene expression and metabolic competency that should be considered when used in cell biology studies and drug toxicity testing.

## Introduction

The liver plays a central role in the detoxification of drug and other xenobiotics and despite its enormous potential for regeneration, morphological and pathophysiological changes induced at toxic doses limit its functions that may result in injury and even acute liver failure. There is unmet need to implement advanced technologies for an early recognition of drug induced liver injury (DILI) and alternative testing strategies based on *in vitro* systems raise the hope to be more informative and to contribute substantially to the reduction of animal use in biomedical research. In recent years, significant advances in high throughput molecular technologies have been achieved, and there has been the recognition that many of these technologies have applications in the detection of risk for DILI and an understanding of drug safety, though implementation has been more difficult than originally envisaged [1,2].

Various hepatoma cell lines have been employed for their ease of handling, unlimited life span and stable phenotype and include the HepG2, HepaRG and HTC cell lines. A major limitation in their use is the fact that they do not faithfully mirror the metabolic activities of healthy liver cells, as exemplified by the impaired activity of drug metabolizing enzymes. The reduced metabolic competency of hepatoma cells like HepG2 can be attributed, in part, to the poor activity of some liver enriched transcription factor (LETF) and a culture system which supports cellular proliferation but not cellular differentiation. In contrast, hepatic metabolism induced by drugs can be studied more reliably in primary cell cultures. Hence, primary cell cultures are widely used and these *in vitro* models should reflect *in vivo* hepatic functions. The advent of 2D culture (collagen sandwich) and three dimensional spheroid culture systems based on components of basement membranes (BM) equipped researchers with biologically more relevant models [3].

In this regard, components of extracellular matrix (ECM) play a vital role in cellular growth, differentiation and metabolic behavior and contribute to cell polarity for proper cell signaling. BMs are thin (50–100 nm) proteinaceous components of ECM which underline the epithelial and endothelial tissues forming a barrier between the cell monolayer and stromal tissue [4]. They provide structural support to cells and modify cellular behavior via outside-in signaling. Although they are ubiquitous in distribution, they show diversity in molecular composition as well as in biological function. The basement membranes are rich in type IV collagen while type I collagen is the most abundant protein in human body [5]. The other major components of basement membrane are laminin, perlecan, heparan sulfate and entactin/nidogen. They are also rich in growth factors (transforming growth factor beta, fibroblast growth factor, epidermal growth factor and platelet derived growth factor) and proteases (matrix metalloproteases, urokinase and tissue plasminogen activator). However, heterogeneity in composition of ECM poses a challenge in constructing tissue specific matrices. Notably, Matrigel, a reconstituted basement membrane extract of Engelbreth-Holm-Swarm (EHS) murine sarcoma is frequently employed as substrata to promote survival and metabolic activity of cells in culture [6]. Hepatocytes plated on Matrigel form smaller aggregates with lower expression of dedifferentiation markers (actin, tubulin, vimentin etc.) [7]. Similarly, hepatocytes when cultured on the synthetic peptide-based hydrogel PuraMatrx^TM^ form three dimensional spheroids/aggregates of different sizes and restore liver functions rapidly while cells cultured on poly-2-hydroxyethyl methacrylate (pHEMA) microcarriers, particularly when coated with collagen and/or fibronectin support hepatocyte attachment and cell spreading. It was also reported that modified alginate sponges overcome limitation of oxygen and nutrition delivery to the center of the spheroid [3]. Several studies have shown the effect of different basement membranes on cell differentiation and polarization [8, 9] and LeCluyse and colleagues [10] reviewed comprehensively current challenges of organotypic liver culture models for toxicity testing. This included an appraisal of the various ECM used in maintaining hepatic structure and function. Over the years, diverse culture systems have been developed to improve hepatocyte morphology and functionality as exemplified by co-culture systems containing supportive non-parenchymal cells such as HepatoPac micropattern [11] and 3D spheroidal culture [12] and detailed information regarding the diverse in vitro models and biomaterials used to facilitate adherence of hepatocyte to culture plates as to restore morphology, function and growth can be obtained from recently published reviews [10, 13–16].

Owing to its importance diverse substrata are used and include collagens, liver biomatrix, Matrigel, collagen-coated plates with an Matrigel overlay to influence hepatocytes functionality [10, 14–15] and it is the pioneering works of Caron [17] and DiPersio and colleagues [18] that were among the first to allude to the importance of extracellular matrix in coordinately modulating activity of liver enriched transcription factors and hepatocyte morphology. For instance, HNF3α expression increases when hepatoma cell lines are cultured on an extracellular matrix gel substratum [19]. Moreover, hepatocytes when cultivated as monolayers supports cell proliferation; however at the expense of cell polarity that is associated with an altered morphology including formation of stress fibers and a reduction in liver specific functions [16]. A typical cuboid phenotype of hepatocytes can be achieved by overlaying the cultures with a collagen gel while the spheroid culture system offers a scaffold for functionally viable hepatocytes with abundant expression of liver-specific transcription factors (HNF4α and C/EBPα) and downstream target genes (albumin, tryptophan 2,3-dioxygenase, arginase 1 and cytochrome P450 7A1). It is further suggested that the switch in cellular dedifferentiation occurs easily on monolayer culture [20] whereas the spherical phenotype of FLC-4 cells is associated with a markedly enhanced expression of genes responsible for drug and lipid metabolism through up-regulation of HNF4α when cultured on a type I collagen-coated plastic dishes [21].

In general, the molecular interaction between HNF4α and hepatocyte specific genes is via protein-DNA and mRNA—microRNA interactions and expression of CYP3A1 and CYP1A2 are more stable or even higher (CYP2B2) in 3D culture on collagen-coated nanofibrous scaffolds than 2D cultured hepatocytes treated with 3-methylcholanthrene and dexamethasone. Additionally, a slight decrease in albumin expression was reported in 2D culture systems [22] while the study of Tuschl and Mueller [23] evidenced transcript expression of CYP2C and CYP4A1 to be decreased after 3 days of rat hepatocytes cultured on serum-containing monolayer as compared to serum free monolayer cultures.

Despite significant advances in biomaterials and cell culture protocols the role of matrix components in the regulation of LETFs and the complex pathways initiated by these factors have not been studied in detail.

Specifically, LETFs are trans-acting DNA binding proteins and are indispensable for expression of hepatic lineage marker and to commit embryonic stem cells to differentiate into hepatocyte [24]. Furthermore, ECM modifies cell-cell and cell-matrix interaction to influence liver gene expression. However, as of today the effects of ECM on the activity of LETF has not been studied in detail. Therefore, we aimed at investigating the effect of two common ECM substrata on the expression and DNA binding activity of LETF and their target hepatic lineage markers in cultures of primary rat hepatocytes and to link this information to cellular morphology and metabolic function. Moreover, the metabolic competency of hepatocytes cultured on different ECM was studied by determining metabolism of testosterone and its metabolites. We also studied the effects of Aroclor 1254, i.e. a mixture of polychlorinated biphenyls, known for its ability to induce drug metabolism and to modulate hepatic gene expression in rat hepatocytes cultured on different ECM and to compare the results with findings obtained after *in vivo* treatment of male Sprague-Dawley (SD) rats with Aroclor 1254. Overall, this study aimed at investigating the interplay between ECM and the complex regulatory cascades of LETF governing the metabolic competency of primary hepatocytes in cultures.

## Materials and Methods

### Animals

Formal approval to carry out animal studies was granted by the animal welfare ethics committee of the State of Lower Saxony, Germany (‘Lower Saxony State office for Consumer Production and Food Safety’ (LAVES)). The approval ID is Az: 33.9-42502-04-06/1143. The investigation conforms to the Guide for the Care and Use of Laboratory Animals (The National Academy Press, Washington, D.C., 1996). Male SD rats were obtained from Charles River laboratories (Sulzfeld, Germany). Animals were housed at animal facility of Fraunhofer ITEM, Hannover, Germany with a standard 12 hours of light/dark cycle and ad libitum food and water.

### Preparation of rat tail collagen

Rat tail collagen was prepared as originally described by Elsdale and Bard [25]. In brief, collagen fibers isolated from rat tails were washed twice with 150ml 1% NaCl and 200ml of distilled water. Subsequently the fibers were suspended in 800 ml of 3% acetic acid solution and stirred overnight at 4°C. The resulting mixture was filtered through sterile gauze and centrifuged for 2 h at 8600 rpm for 15 min at 4°C. The volume of the supernatant was determined and 1/5th of the total volume was added as 30% NaCl solution overnight at 4°C. The collagen was precipitated by centrifugation at 5000 rpm for 35 min at 4°C. The pellet was washed once with 100 ml of 5% NaCl+0.6% acetic acid and re-suspended in 200 ml of the same solution and stirred overnight at 4°C for homogenization. Subsequently, collagen was poured into the dialysis tubes. The hoses were stirred in a beaker with 1 mM HCl at 4°C. The acid was changed for five times and the total protein was determined according to Smith et al. [26].

### Isolation of rat hepatocytes

Rat hepatocytes were isolated according to the original method of Seglen [27] and modifications reported in [28–32]. Briefly, after laparatomy, the vena porta was prepared, cannulated and perfused with calcium-free Krebs—Ringer buffer (KRB) until visibly lightened. Subsequently, the liver was perfused with 100ml of 1mM EDTA buffer followed by a 0.5% collagenase-KRB solution. The liver was transferred to ice-cold wash-buffer and after removal of the liver capsule with anatomical forceps hepatocytes were obtained by gently shaking of the organ. The hepatocyte suspension was filtered through nylon mesh (100 mm) to remove debris and washed two times with the washing buffer (1000 ml Hank’s balanced salt solution (PAA, Germany) supplemented with 2.4 g Hepes (Sigma) and 2 g bovine serum albumin (Sigma) followed by centrifugation at 4°C and 500 rpm for 5 min. The resulting pellet was re-suspended in William’s E medium (Biochrom, Berlin, Germany) supplemented with 5% fetal calf serum (FCS, Biochrom), 9.6 μg/ml prednisolone, 0014 μg/ml glucagon (Novo, Germany), 0,16 U/ml insulin (Hoechst, Germany), 200 U/ml penicillin, and 200 U/ml streptomycin (GIBCO, Germany). The cell suspension was adjusted to 1 million cells per ml. Before seeding, the viability of cells was determined by the trypan blue exclusion test and found to be > 85%. A total of 2 ml of the hepatocyte suspension was used for cell seeding on collagen coated culture dishes (see below). Twenty-four hours after seeding, cultures were overlaid with a second collagen layer as originally described by Dunn et al. [33]. Medium was changed once daily using William’s E medium containing 5% FCS.

### Culture of primary hepatocytes as collagen sandwich

Collagen coating of 60 mm petri dishes (BD Biosciences, Heidelberg, Germany) was done as previously described with minor modifications [31]. The pH of the collagen was adjusted to 7.4 using a NaHCO3 solution and 1 ml of a 0.5 mg/ml collagen solution was applied to coat the 60 mm Petri dishes (Greiner, Frickenhausen, Germany). The dishes were prepared in advance of cell isolations. Following seeding of 2 x 106 hepatocytes per dish the cells were allowed to attach. After 24 h in culture, the medium was removed along with non-adherent cells. A second layer of collagen was pipetted on top of the cells. After gelation of the second layer culture media [2 ml of William’s E supplemented with 5% fetal calf serum (FCS; Biochrom), 9.6 μg/ml prednisolone, 0014 μg/ml glucagon (Novo, Germany), 0,16 U/ml insulin (Hoechst, Germany), 200 U/ml penicillin, and 200 U/ml streptomycin (GIBCO, Germany)] was added and changed daily. The cells were cultured at 37°C and an atmosphere of 5% CO2 and were inspected by phase contrast microscopy every 24h.

### Culture of primary hepatocytes on Matrigel

Coating of 60 mm petri dishes with BD Matrigel (10 mg/ml, BD Biosciences, Heidelberg) was done by adding 980 μl of 1:2 diluted Matrigel solution in Ham's F12 medium (Biochrom, Berlin, Germany). The coated petri dishes were stored for 1 h at 4°C followed by 37°C for 30 min for polymerization. Seeding density of hepatocytes, cell culture conditions and type of media (Williams E with supplements, changed daily) was the same as described for the collagen sandwich cultures. Every 24h the cells were inspected by phase contrast microscopy.

### Aroclor 1254 treatment of hepatocyte cultures and of SD rats

Following a recovery period of four days after seeding, the cultured hepatocytes were treated with 10 μM of Aroclor 1254 for 24 hours. The final concentration of DMSO (solvent for Aroclor 1254) in culture medium was < 0.3% v/v. In order to compare in vitro results with in vivo findings, male SD rats (n = 5, average body weight 230 gr) were given a single i.p. injection of 100 mg/kg body weight Aroclor 1254 dissolved in corn oil. Seventy two hours after this single treatment RNA was isolated from rat liver while untreated rat liver served as control. Note, previous studies had demonstrated that at the 72h time point the administered Aroclor 1254 dose was completely absorbed and therefore this time point is more appropriate for in vitro-in vivo comparisons.

### Reverse transcription-polymerase chain reaction (RT-PCR)

Total RNA was isolated from the rat hepatocytes cultured on the two matrices on 5, 6, 7, 9 and 12 days post isolation (Qiagen RNeasy Kit). The concentration and quality of isolated RNA was checked by measuring absorbance at 260/280 nm and electrophoretic separation on 1.5% agarose gel. Subsequently, cDNA was prepared from the isolated RNA using Omniscript RT kit (Qiagen, Germany). The polymerase chain reaction (PCR) was carried out to amplify cDNA using 25 ng of each cDNA in 19 μl reaction mixture containing 3 U of Taq DNA polymerase (Qiagen, Hilden Germany), 10 x reaction buffer (2 μl), 10 mM dNTPs (0.5 μl) and 4 μM of gene specific primers (5 μl). Amplification was carried out for 35 cycles each, at 94°C for 30 sec, 54°C for 45 sec, 72°C for 1 min with a final extension at 72°C for 5 min in a thermal cycler (T3, Biometra, Göttingen, Germany). The PCR product was separated on 1.5% agarose gel containing 5 μg/ml of ethidium bromide and quantified by densitometric analysis (Kodak ID image analysis software). Note, the expression of housekeeping genes β2 microglobulin and 18S rRNA remained unchanged and gene expression data was subsequently normalized to the mean expression level of internal controls. The primers used are listed in S1 and S2 Tables.

### Isolation of nuclear extracts from collagen culture

Cultured cells were washed twice with 2 ml of cold PBS and lysed using 1 ml of Nonidet-P40 lysis buffer (150 mM NaCl, 1% NP-40 and 50 mM Tris, pH 8). The lysate was vigorously resuspended and incubated on ice for 15 min and filtered through cell strainers (BD Biosciences, Heidelberg, Germany). The cell sieves were washed thrice with 1 ml of lysis buffer and the filtrate was centrifuged in a Megafuge at 4°C, 2000 rpm for 5 min. The pellets were resuspended in 300 μl of lysis buffer and a sucrose cushion was loaded and centrifuged in a microfuge at 4°C and 14000 rpm for 10 min. The pellets were added to 3 μl of Dignam C buffer (0.5M EDTA 40 μl, 25 ml Glycerin, 2 ml Hepes 1M pH 7.9, 150 μl MgCl2 1M, 21 ml NaCl 2M, 1 μl DTT 1M, 40 μl β-Glycerophosphate 1M 5 μl sodium orthovandate 200 mM) (+)/dish and again centrifuged for 10 min at 4°C and 14000 rpm. The volume of the supernatant was determined and 30 μl of nuclear extract was aliquoted and snap frozen in liquid nitrogen and subsequently stored at -80°C.

### Isolation of nuclear extracts from Matrigel culture

Cells were washed with 2 ml of cold PBS and then with 3 ml of MatriSperse Cell Recovery Solution (Biotech, BD Biosciences, Germany) at 4°C. Following this, cells were scrapped using 1.5 ml of Matrisperse (Cell Recovery Solution) and cell suspension was incubated for one hour on ice followed by centrifugation at 200–300xg (1100 rpm) for 5 min at 4°C. The pellet obtained was washed twice with 40 ml of cold PBS and again centrifuged for 5 min at 200–300xg (1100 rpm) at 4°C. The pellets was dried carefully and snap frozen in liquid nitrogen and subsequently stored at -80°C. For preparation of nuclear extracts, 4 ml of 1% sodium NP40 lysis buffer was added to pellet for 30 min on ice. The lysate was centrifuged for 5 min, 2200 rpm and 4°C. The pellets were resuspended in 300 μl of NPB and 400 μl of sucrose (50%) was added. The second steps onwards are similar to collagen culture.

### Western blotting of LETFs

Western blotting of LETFs was done as previously described [34–39]. Expression of LETFs in rat hepatocytes cultured on collagen and Matrigel was studied on day 7, 9 and 12 and in the case of HNF4α and HNF-6 on days 5, 6 and 7. Approximately, 38 μg (HNF1α, HNF1β, HNF3α, HNF3β and HNF3γ), 20 μg (HNF4α and HNF6) and 45 μg (C/EBPα and C/EBPβ) of isolated proteins were separated on 12% SDS-PAGE gel (60–90 min at 25mA) and subsequently transferred to PVDF membrane (0.2 μm, NEN) following the manufacturer’s recommendation (2 h at 350 mA). The membrane was blocked for 1h at RT with TBS- Tween 20 (TBST) containing 5% nonfat dried milk. Then, washed 3x for 5 min each and incubated with the respective primary antibody dissolved in TBST for 1h at RT and subsequently washed thrice with washing buffer. The membrane was incubated with an appropriate secondary antibody (1:10,000) for 1h at RT and washed thrice with washing buffer. Following this, the membranes were incubated for 1 min in 3 ml of chemiluminescence reagent (NEN) and signals were detected with Kodak imager station 440CF. Quantification of band was performed using the LI-COR image studio Lite software. Note, rat liver nuclear extract was prepared as originally described by Gorski et al. [40] and Niehof and Borlak [39]. The nuclear extract were frozen in aliquots and stored at -80C. Typically, 40 μg was taken as positive control for all the western blot experiments. Details of the primary and secondary antibodies used are given in S3 Table.

### Western blotting of CYP monooxygenases

On day five in culture protein expression of CYP monooxygenases was studied. For this purpose primary rat hepatocytes cultured on Matrigel and collagen were lysed using the RIPA buffer (50 mM Tris HCl pH 8, 150 mM NaCl, 1% NP-40, 0.5% sodium deoxycholate and 0.1% SDS) and Western blotting experiments were performed as described above with 30 μg of each protein. Rat liver extract (40 μg) was taken as positive control. The details of primary and secondary antibodies used are given in S3 Table.

### Electrophoretic mobility band shift assay (EMSA)

The nuclear extract (2.5 μg) and 105 cpm (0.027 ng) of ^32^P labeled oligonucleotides (MWG Biotech, Germany) were incubated in binding buffer (25 mM Hepes, pH 7.6, 5 mM MgCl_2_, 34 mM KCl, 2 mM DTT, 2 mM Pefabloc, 2% v/v aprotinin, 40 ng of poly (dI-dC)/μl and 100 ng of bovine serum albumin/μl) for 20 min on ice. Free DNA and DNA-protein complexes were resolved on a 6% polyacrylamide gel. Supershift experiments were done with specific antibodies. Gels were blotted to Whatman 3 MM paper, dried under vacuum, exposed to imaging screens for autoradiography and analyzed using a phosphor imaging system (Molecular Imager FX pro plus; Bio-Rad Laboratories GmbH, München, Germany). Gene specific antibodies and probes used are listed in S3 and S4 Tables, respectively.

### Testosterone metabolites identified by HPLC

Cultures of primary hepatocytes were treated with 100 μM testosterone (dissolved in William's Medium E) on days five and eight post isolation and cultivation. After 24 h of substrate incubation the cell culture supernatant was collected and testosterone and six of its metabolites (6α-hydroxytestosterone, 7α-hydroxytestosterone, 6β-hydroxytestosterone, 16α-hydroxytestosterone, 2α-hydroxytestosterone and androstenedione) were detected by HPLC with 11α-hydroxyprogesterone as the internal standard as previously reported [31].

### Detection of ECM components by 2D gel electrophoresis and MALDI-TOF-MS

The molecular composition of Matrigel and rat tail collagen was determined by 2D gel electrophoresis and MALDI-TOF-MS (Bruker Daltonics). The protein content of the collagen and Matrigel matrix was 0.5 mg/mL (in 1mM HCl solution) and 11.1 mg/mL respectively. Subsequently, the Matrigel sample was diluted and the collagen sample was lyophilized and adjusted to a concentration of 5 mg/ml. One mg each was used to perform 2D gel electrophoresis. The separated protein spots on gels were digested with trypsin and identified by MALDI-TOF-MS following the method described by Garaguso and Borlak [41].

### Statistical analysis of gene expression data

Statistical analysis was performed with the SPSS software version 21. Analyses of variance (ANOVA) was estimated to explore the effects of extracellular matrix substrata and time on the expression and DNA binding activity of LETF and their target hepatic lineage markers in cultures of primary rat hepatocytes by means of a multi-factorial model (factors: time, matrix, gene and/or protein expression). Notably, ANOVA testing did not reveal a statistically significant time effect. Hence, an independent T-Test was performed to compare the effects of extracellular matrix substrata on the variables gene expression using the SPSS software and the results were considered significant at p values of ˂ 0.05. For both ANOVA and the T-Test Bonferroni post hoc testing was performed. Unless otherwise stated n = 5 independent experiments were considered.

## Results

### Morphology of cultured primary hepatocytes

**Fig 1:**
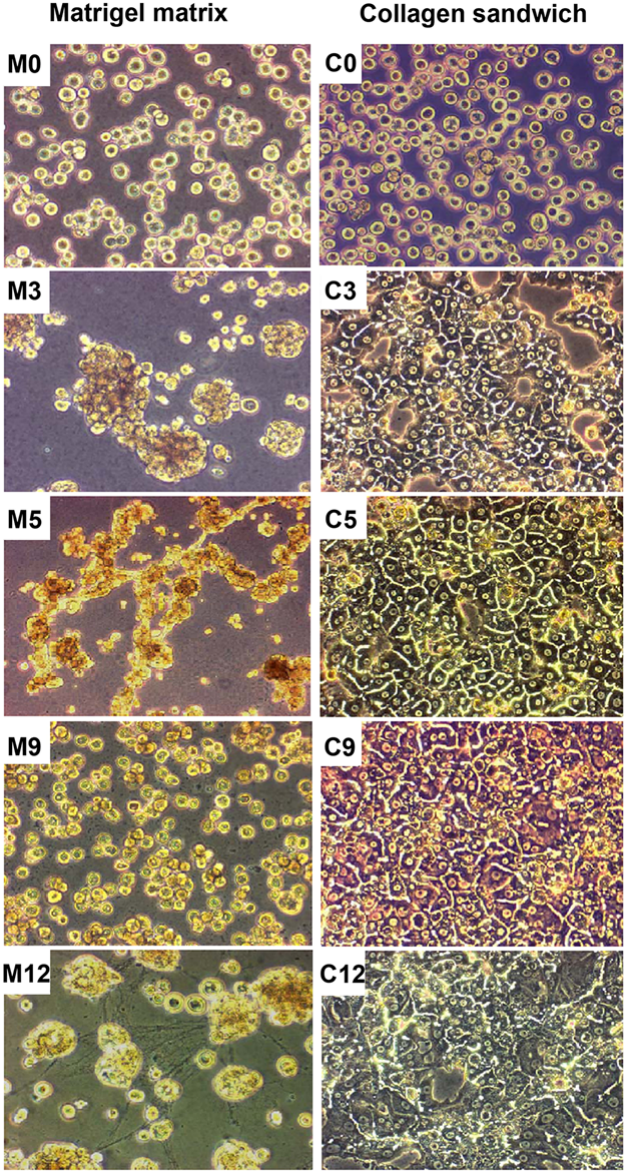
Morphology of primary rat hepatocytes cultured on Matrigel or collagen. Morphological appearance of isolated rat hepatocytes cultured either as collagen sandwich or on Matrigel on day 0 **(M0 and C0)**, day 3 **(M3 and C3)**, day 5 **(M5 and C5)**, day 9 **(M9 and C9)** and day 12 **(M12 and C12)** of culture. Images are presented at 200x magnification. M: Matrigel C: Collagen.

Cultures of primary rat hepatocytes grown on rat tail collagen and Matrigel were examined daily by phase contrast microscopy. The cells displayed divergent morphologies as early as on the second day of seeding. After adhering to rat tail collagen, hepatocytes started spreading and developed polygonal structures with easily identifiable cell boundaries (Fig 1C3). The cells were often binucleated. The confluency of the cultures in all repetitions was around 85–90%. During later stages, the hepatocyte cytoarchitecture and cell polarity appeared organotypic and formation of bile canaliculi was suspected (Fig 1C5). An increased number of dead and detached cells were noticed from the ninth day of culture (Fig 1C9). The adherent cells begun granulating and around 70% of the cells were detached on the twelfth day (Fig 1C12). In contrast, hepatocytes cultured on Matrigel formed aggregates. Morphological inspection revealed small foci (Fig 1M0 and 1M3) and a filamentous structure of cells that was noted between them on the fifth day of culture thus suggesting significant changes to the cytoskeleton (Fig 1M5). Further with time, these structures disintegrated and again the cells formed smaller clusters (Fig 1M9 and 1M12).

### Gene expression of liver specific transcription factors and their target genes

To determine the effects of extracellular matrix on the activity of LETFs and some hepatic lineage markers hepatocytes were cultured on either Matrigel or collagen. Initially, transcript expression of different housekeeping genes was considered and a statistically significant difference in expression was noticed for GAPDH (p<0.015) and 28S rRNA (p<0.000) while transcript expression of β2 microglobulin (p<0.190) and 18S rRNA (p<0.661) remained unchanged (S1 Fig). However, after Bonferroni adjustment for multiple testing none of the house keeping genes differed significantly. Furthermore, ANOVA testing did not reveal a statistically significant time effect. Note, gene expression changes were considered on days 5, 6, 7, 9 and 12 in culture and it did not matter whether the ANOVA was performed independent of the housekeeping gene 18S rRNA or by considering gene expression changes relative to 18S rRNA.

Thus, the gene expression of HNF1α, HNF3β, HNF3γ, HNF4α, C/EBPα and C/EBPβ was similar for hepatocytes cultured on either matrices. In contrast, HNF1β (p<0.001), HNF3α (p<0.031), HNF6 (p<0.005), C/EBPγ (p<0.037) and C/EBPδ (p<0.047) were up-regulated in hepatocytes cultured on collagen as compared to Matrigel. Furthermore, Coup-TF I that competes with HNF4α for its cognate recognition site did not differ in transcript expression when hepatocytes were cultured on either substratum. However, a significant change in CDP gene expression was noticed for hepatocytes cultured on collagen (p<0.049). Applying the Bonferroni correction only HNF1β remained significantly different (p<0.001) in transcript expression amongst the two culture systems.

In order to determine the effect of LETFs on target genes their expression pattern in cultured hepatocytes were studied. For albumin and enzymes of energy metabolism (G6P, G6PDH and PEPCK), expression was not affected by the type of ECM. In contrast, OTC gene expression was significantly increased (p = 0.035) with cells cultured on Matrigel, however did not remain significant after Bonferroni adjustment. Taken collectively and despite the significant difference in morphological appearance (see Fig 1) most of the LETFs and several of their target genes did not differ in transcript expression when cultured on either collagen or Matrigel.

### Western blotting of LETFs

**Fig 2:**
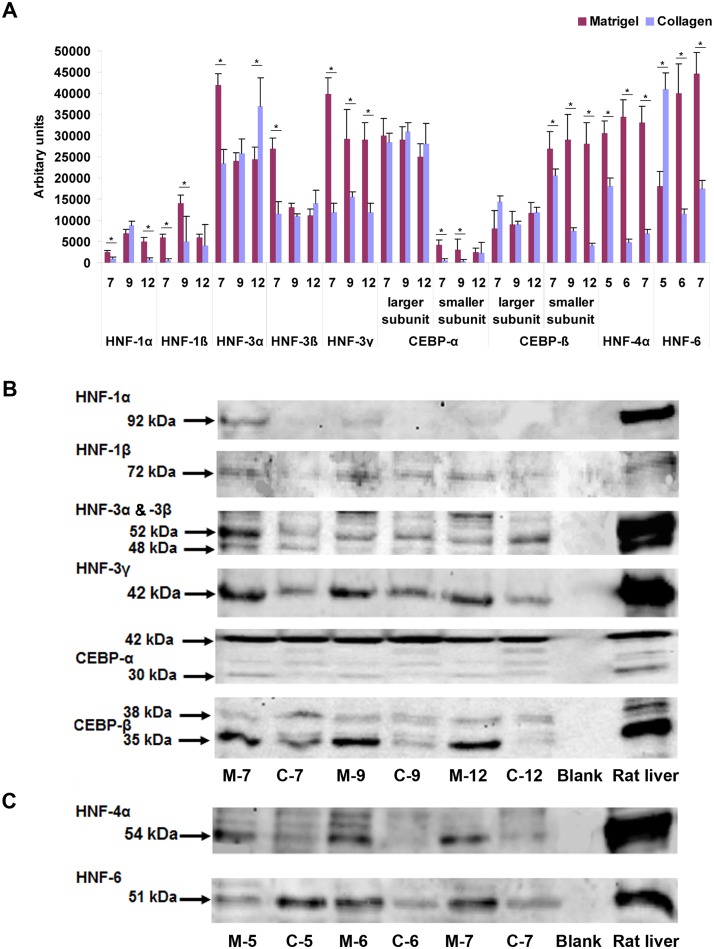
Western blotting of liver enriched transcription factor in rat liver and primary rat hepatocytes cultured on Matrigel and collagen. **(A)** The relative band density was determined using LI-COR image studio of the Lite software. Expression of HNF-1α, HNF-1β, HNF-3γ, HNF-4α, HNF-6 and smaller isoforms of C/EBP-α and C/EBP-β (p30 and p35, respectively) was higher in cells cultured on Matrigel as compared to collagen sandwiched hepatocytes. **(B and C)** Image of Western blot of liver enriched transcription factors. Isolated proteins were separated on 4–12% SDS-PAGE and transferred onto a PVDF membrane as described in the Material and Method section. M: Matrigel, C: Collagen.

A representative example of n = 3 independent Western blot experiments is depicted in Fig 2 and the bands were quantified as described in the Material and Method section. Although not always consistent, expression of HNF1α, HNF1β, HNF3γ, HNF4α and HNF6 was mostly increased with hepatocytes cultured on Matrigel as compared to collagen sandwiched hepatocytes. For instance, a higher expression of HNF3α, HNF3β, HNF3γ, HNF4α and HNF6 was observed with hepatocytes cultured on Matrigel for 7 days (Fig 2A–2C). Furthermore, the larger isoforms of C/EBPα and C/EBPβ (p42 and p38, respectively) were equally expressed in hepatocytes cultured on either matrices. Thus, the C/EBPα larger subunit served as an internal loading control. Nonetheless, the smaller isoforms (p30 and p35, respectively) of both TFs were significantly up-regulated with hepatocytes cultured on Matrigel as compared collagen sandwiched hepatocytes (Fig 2A–2C). The Western blotting of LETFs of hepatocytes cultured on collagen suggests a time dependent decline in expression of the smaller subunit of CEBPß and HNF1α on day 12 and for HNF4α and HNF6 on day 7, respectively. Conversely, the protein expression of HNF1ß and HNF3α was increased over time for hepatocytes cultured on collagen. Lastly, with hepatocytes cultured on Matrigel the expression of HNF1ß, HNF3α, ß and γ was reduced in expression over time, while that of HNF4α and HNF6 increased with culture time.

### MALDI-TOF-MS analysis of collagen and Matrigel substrata

The proteins of cell free samples of the two ECM matrices were analyzed by 2D gel electrophoresis and MALDI-TOF mass spectrometry. Amongst the protein identified, collagen was common to both of the matrices. Laminin and nidogen (entactin) were the major ECM component detected in Matrigel while the cytoskeleton and filament related proteins were detected in rat tail collagen (Tables 1 and 2). Note, a quantitative analysis was not attempted.

**Table 1:** List of non-common proteins identified in cell free samples of Matrigel by MALDI-TOF-MS analysis.

Sn	Number	Gene	M.wt (kDa)	Protein	Function
1	gi|6678656	Lama1	338.2	Laminin-α-1	ECM-component, glycoprotein cell-matrix-adhesion, receptor association, signal transduction regulation of adhesion and migration
2	gi|126367	Lamb1-1	196.8	Laminin β1 Precursor, Laminin γ1	ECM-component, cell matrix adhesion, EGF-analog domain
3	gi|6754854	Nid1	136.5	Nidogen 1(Entactin)	ECM-component, calcium binding, cell matrix adhesion, ECM crosslinking, Nidogen precursor
4	gi|34810643		30.0	A Chain A	Nidogen Laminin complex, ECM-component, cell matrix adhesion
5	gi|31981722	Hspa5	72.4	Heat shock protein 5	Chaperon in endoplasmic reticulum, binding with transthyretin, glucose regulation

**Table 2:** List of non-common proteins identified in cell free samples of rat tail collagen by MALDI-TOF-MS analysis.

Sn	Number	Gene	M.wt (kDa)	Protein	Function
1	gi|9845234	Anxa2	38.7	Annexin A2	Plasminogen receptor, cytoskeleton associated, organisation of collagen fibers; phospholipase inhibitor-activator
2	gi|42558981	Ppfia2	143.2	Liprin-α-2	Protein tyrosine phosphatase receptor type f (PTPRF) polypeptide interacting protein alpha-2, cellular localisation of PTPRF and focal adhesion
3	gi|20664363	1LCU_B	41.3	B Chain B	Anti-parallel actin dimerization, actin filament polymerisation
4	gi|229690	1ATN_A	41.6	A Chain A	Endo-desoxyribonuclease-I in complex with actin
5	gi|11603008	CAST	49.1	CalpastatinType IV-L	Calpain Inhibitor
6	gi|21357425	Krt 20	57.1	Keratin complex 2	Intermediate filament protein

### DNA binding activity of LETFs

**Fig 3:**
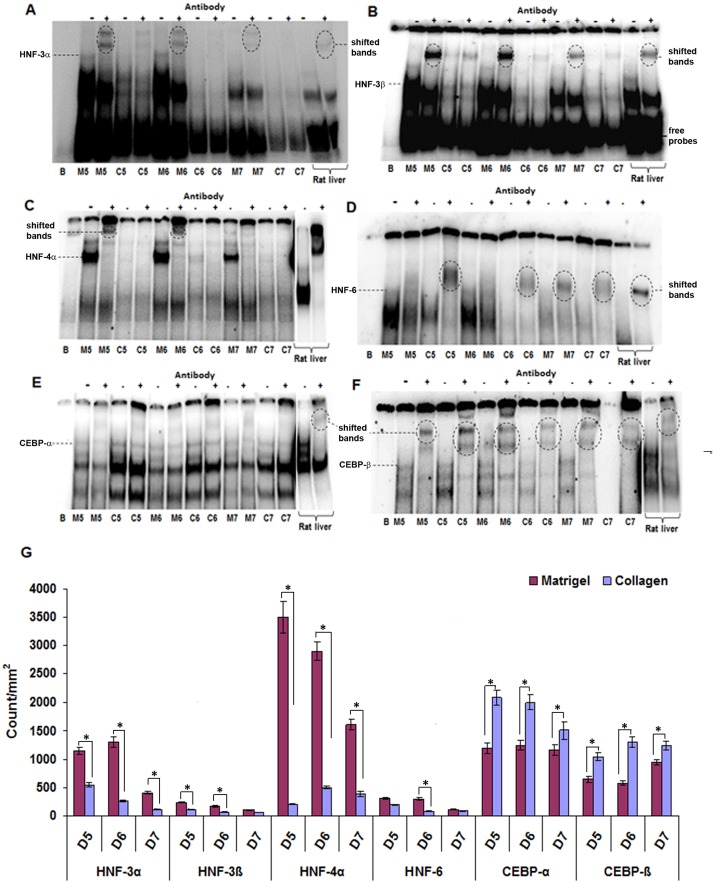
DNA binding activity of liver enriched transcription factors in primary rat hepatocytes cultured on Matrigel or collagen. EMSA assays for HNF-3α **(A)**, HNF-3β **(B)**, HNF-4α **(C)**, HNF-6 **(D)**, C/EBP-α **(E)** and C/EBP-β **(F)** were carried out with nuclear extracts isolated on day 5, 6 and 7 of culture. The results revealed that the binding activity of HNF-3α, HNF-3β, HNF-4α and HNF-6 to be higher when cultured on Matrigel whereas the opposite was seen for C/EBP-α and -β when rat hepatocytes were cultured as collagen sandwich. Nuclear extracts of rat liver served as positive control for EMSA assays. The results are given in count/mm^2^ **(G)**. B: Blank, M: Matrigel, C: Collagen.

The DNA binding activity of HNF3α, HNF3β, HNF4α and HNF6 was considerably higher in cells cultured on Matrigel (3.4, 2.6, 7.5 and 1.8 fold, respectively) at all-time points studied (Fig 3A–3D and 3G). In contrast, C/EBPα and C/EBPβ binding activity (1.6 and 1.7 fold, respectively) of cells increased marginally when cultured on rat tail collagen (Fig 3E–3G). With the exception of HNF4α on collagen, the DNA binding activity of the transcription factors declined with time irrespective of the ECM type. Note, the depicted images are representative of n = 3 independent band shift assays.

For comparison, band shift assays for HNF 1–4 and C/EBP-α with rat liver nuclear extracts are shown in S2 Fig and DNA binding activity of all LETFs investigated could be demonstrated.

### Western blotting of CYP monooxygenases

**Fig 4:**
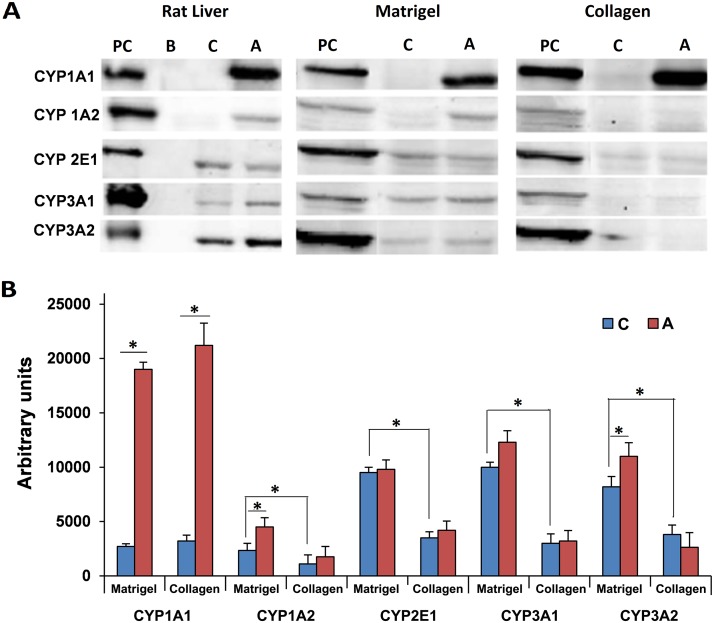
Expression of CYP enzymes in primary rat hepatocytes cultured on Matrigel or collagen. **(A)** Depicted are representative Western immunoblots of protein extracts of rat hepatocytes cultured for 5 days. **(B)** The relative band density was determined using LI-COR image studio of the Lite software. Data are mean±SD of n = 3 independent experiments. PC: positive control (rat liver extract); B: blank lane; C: control hepatocyte cultures; A: Aroclor 1254 treated cultures

We previously investigated transcript expression of major cytochrome P450 monooxygenases in cultures of rat hepatocytes [29] and based on our earlier findings prominent members were selected for Western blotting studies. This revealed expression of CYP1A2, CYP2E1, CYP3A1 and CYP3A2 to be significantly increased in hepatocytes cultured on Matrigel when compared to collagen sandwiched cultures. However, expression of CYP1A1 was comparable in the two culture systems tested. Furthermore, treatment with Aroclor 1254 significantly increased the expression of CYP1A1 on Matrigel and collagen cultured hepatocytes thus evidencing similar responsiveness of both culture systems to this inducer (Fig 4A and 4B).

### Testosterone metabolism

**Fig 5:**
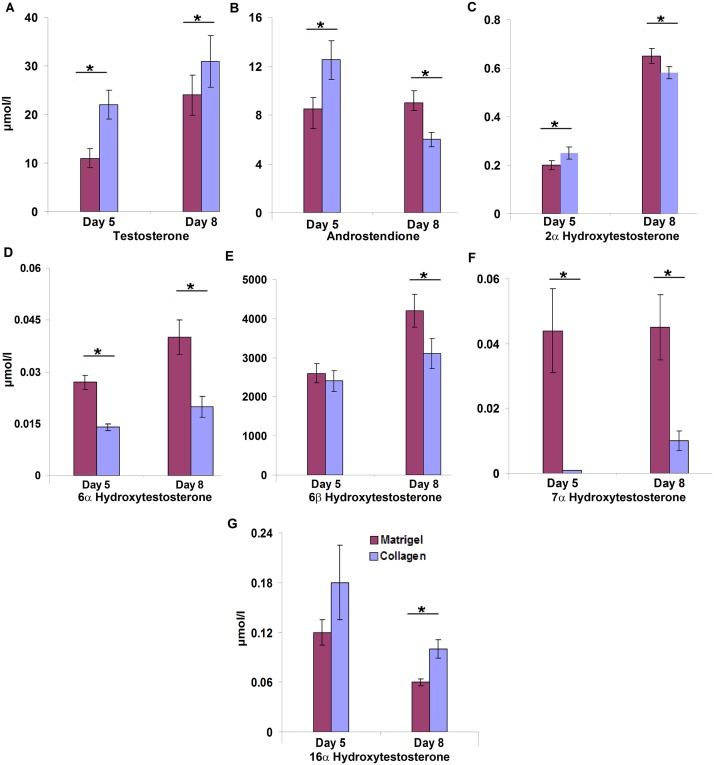
Testosterone metabolism in rat hepatocytes cultured on Matrigel or collagen. The metabolic competence of rat hepatocytes cultured for 5 and 8 days was assayed at 100 μM testosterone substrate concentration. Metabolites were determined by HPLC in culture media 24h after single testosterone treatment. Data are given as mean±SD of at least three independent experiments. Statistical significance was determined with the Student’s *T*-test.

To further determine the metabolic competency of hepatocytes cultured on different matrices, 100 μM of testosterone was added and a total of six metabolites were detected after a 24 h incubation period. Essentially, less testosterone was metabolized at the fifth (p = 0.001) and eighth (p = 0.021) day of cells cultured of rat tail collagen (Fig 5A). Conversely, production of androstendione, i.e. the most abundant metabolite, was significantly higher on Matrigel cultured hepatocytes on day eight (p = 0.001). However, no significant difference in androstendione production in either of the culture systems was observed on the fifth day of culture (Fig 5B). Furthermore, production of 2α-hydroxytestosterone was significantly increased with hepatocytes on collagen at day five in culture while its production increased significantly on day eight (p = 0.006) when cultured on Matrigel (Fig 5C). Likewise, 6α and 7α-testosteronehydroxylase activity was significantly increased on Matrigel cultured cells at both time points (Fig 5D). However, the production of 6β-hydroxytestosterone did not differ amongst the culture systems at the time points examined (Fig 5E and 5F). Finally, production of 16α-hydroxytestosterone increased significantly on day eight (p = 0.002) on collagen cultured cells (Fig 5G).

### Gene expression and DNA binding activity of liver specific transcription factors and CYP monooxygenases in response to Aroclor 1254 treatment

**Fig 6:**
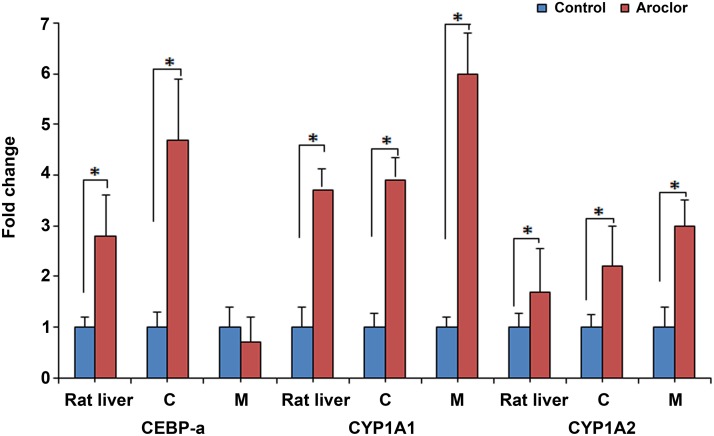
Effect of Aroclor 1254 treatment on expression of liver enriched transcription factors and CYP enzymes in rat liver and rat hepatocytes cultured on Matrigel or collagen. An average of three independent measurements was taken and results are presented as mean fold change +/- SD over control.

The effects of Aroclor 1254 treatment on the gene expression of LETFs and CYP monooxygenases were determined *in vivo* and *in vitro* and the results represent n = 5 independent experiments. Aroclor 1254 treatment caused a significant increase in C/EBPα expression (4.7 folds) in hepatocytes cultured on collagen. Conversely, a marginal decrease of 1.3 fold was determined on Matrigel cultured rat hepatocytes. Additionally, n = 5 rats were treated with a single dose of 100 mg/kg of Aroclor 1254 and sacrificed three days later and this treatment caused a 2.8 fold increase in expression of C/EBPα and induction of CYP1A1 and CYP1A2 mRNA by 3.7 and 1.7 fold, respectively, as compared to controls (Fig 6).

Furthermore, Aroclor 1254 treatment induced significantly expression of CYP1A1 (4 and 6 fold) and CYP1A2 (2.5 and 3 fold) in rat hepatocytes cultured on collagen and Matrigel, respectively. However, the Aroclor 1254 treatment did not result in significant changes in transcript expression of HNF1α, HNF3γ, HNF4α, CYP2E1, CYP3A1, CYP4A1 and of phenyl dehydrogenase, apolipoprotein C III and cyclophilin in either culture systems (data not shown). Note, while these findings generally confirm results from our previous study [29] the difference in CYP3A1, CYP4A1, HNF1 and HNF4 gene expression is likely caused by the different treatment conditions employed, i.e. 24h in the present study versus 48h in the previous study. Likewise, the primers used in the previous study did not distinguish amongst isoforms of HNF1 and HNF4 transcription factors.

**Fig 7:**
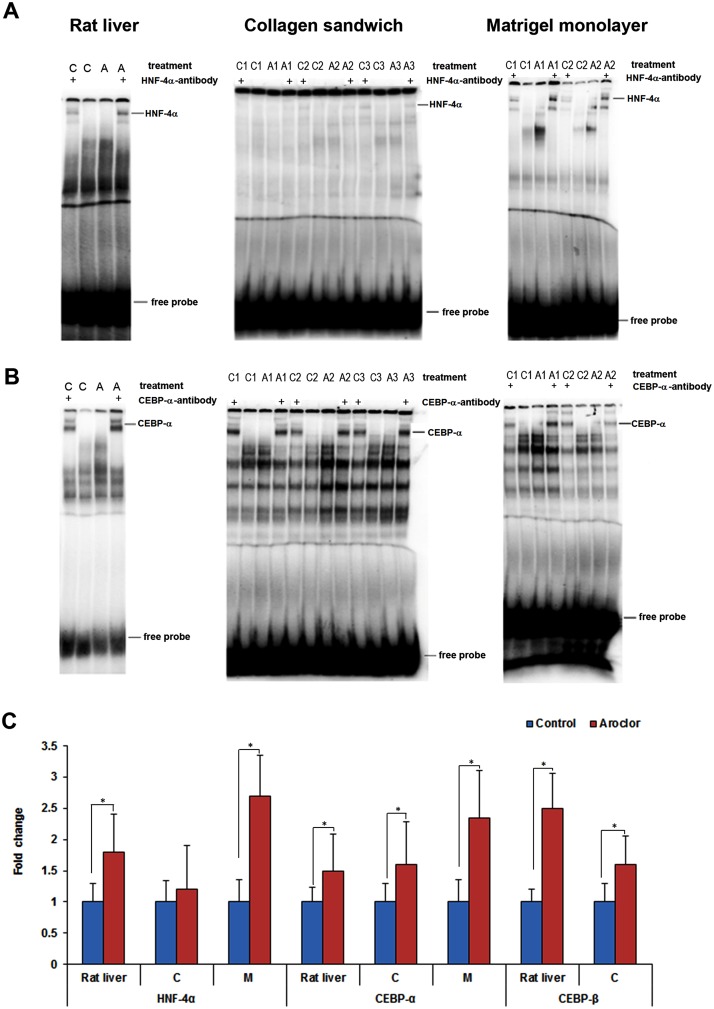
DNA binding activity of HNF-4α, C/EBP-α and C/EBP-β in response to Aroclor 1254 treatment. Representative EMSA images are depicted in panel **(A and B)**. The results are given as fold changes over controls and are shown in panel **(C)**. Band shift with specific antibodies are indicated by the symbol ‘+’. C: Control, A: Aroclor 1254 treated.

Moreover, Aroclor 1254 treatment increased the DNA binding activity of C/EBPα by 1.4, 1.5 and 2.2 fold in rat liver, collagen and Matrigel cultured rat hepatocytes, respectively. Binding activity of C/EBPβ increased by 2.4 and 1.5 fold in rat liver and on collagen cultured rat hepatocytes, respectively. Similarly, 1.6, 1.2 and 2.6 fold increases in DNA binding activity of HNF4α were observed in rat liver, collagen and Matrigel cultured hepatocytes upon Aroclor 1254 treatment (Fig 7A–7C).

## Discussion

Primary hepatocyte cultures are indispensable tools for hepatotoxicity testing. However, during the process of tissue disaggregation and cell isolation, the cells become traumatized that leads to cellular stress and a decline in metabolic competency. A perfect culture system should provide conditions to restore cellular functions which also depend on proper cell-cell and cell-matrix interactions. The use of the collagen sandwich culture system provides conditions to ensure well-differentiated phenotype of hepatocytes and to reestablish cell polarity [42] as was seen in the present study with cultures of hepatocytes being well-differentiated and possibly even forming bile canaliculi, as observed previously [43]. In contrast, cells cultured on Matrigel showed limited cell-matrix adhesion, formed cell aggregates and retained spherical in cellular morphology. These morphological features are often described in connection with tumor cells. Indeed, Matrigel is an extracellular matrix of tumor origin; the observed morphology suggests the importance of matrix component in cellular morphology and growth [44, 45].

Importantly, ECM components communicate to the cytoskeleton through transmembrane receptors like integrin, β-catenin and the hyaluronic acid receptor to influence cellular morphology by controlling the polymerization and organization of the cytoskeleton [46]. This cross talk is carried beyond the cytoplasm to affect expression of genes coding for cell cycle and metabolism amongst others. The role of extracellular signals in regulating liver-enriched transcription factors during hepatocyte differentiation has been the subject of several reviews [47–49] and despite significant advances in the development of improved in vitro systems the regulation of liver enriched transcription factors on divergent ECM substratum was not studied in detail. We hypothesized that apart from providing mechanical and structural support the various substrata will influence integrin, proteoglycan and growth factor receptor signalling and as a result cellular differentiation. We therefore investigated the regulation of liver enriched transcription factors in rat hepatocytes cultures on collagen and EHS sarcoma matrices by RT-PCR, Western blotting and EMSA and evaluated the metabolic competency of cell cultures. This revealed a significant up-regulation of 28S rRNA and GAPDH housekeeping genes in collagen cultured rat hepatocytes while β2 microglobulin and 18S rRNA showed no difference and were therefore used for normalization of results of subsequent studies. Gene expression analysis revealed HNF1α, HNF1β, HNF3α, HNF3β, HNF6, C/EBPγ and C/EBPδ to be up-regulated in rat hepatocytes cultured on collagen while HNF3γ, HNF4α, C/EBPα and C/EBPβ were increased in expression in rat hepatocytes cultured on Matrigel. Nonetheless, only HNF1β, HNF3α, HNF6, C/EBPγ and C/EBPδ reached statistically significance. Oda et al. [8] also reported up-regulation of HNF3α on collagen whereas HNF4α, C/EBPα and C/EBPβ were increased on Matrigel cultured cells. Moreover, expression of COUP-TF1 that functionally interacts with HNF4α at targeted promoters was insignificantly up-regulated on Matrigel cell cultures. In a gene expression profiling studies with primary human hepatocytes and human hepatoma cell lines a Matrigel sandwich configuration retained abundant expression of LETFs such as CEBPα and HNF4α [50].

Conversely, expression of TFs at the protein level revealed a different picture and the mean expression (see day 7, 9 and 12 of panel A Fig 2) of HNF1β, HNF3α and HNF3β were comparable amongst the two culture systems. However, the protein expression of HNF1α, HNF3γ, HNF4α and HNF6 was increased with hepatocytes cultured on Matrigel. Note, HNF4α plays a central role in liver metabolism and in the complex interplay of HNFs. Its P2 promoter driven embryonic isoforms HNF4α7–9 are predominant in dedifferentiated liver and pancreatic cells and are also expressed in the early stages of hepatocarcinogenesis but are below the limit of detection in liver of adult healthy individuals and patients with other diseases [51, 52]. We previously reported the regulation of P2 promoter driven HNF4α isoforms in hepatocarcinogenesis and an identification of novel disease candidate genes found to be regulated in an EGF-induced mouse liver cancer model and human HCC [38].

Besides, the smaller subunits of both C/EBPα and C/EBPβ were more abundantly expressed in Matrigel. However, the larger subunits were found to be comparable in expression in both of the culture systems. Difference in the translation initiation process involved in production of different N-terminally truncated isoforms of C/EBPα and C/EBPβ have been reported [49].

The binding of transcription factors to their cognate recognition sequences is a prerequisite for expression of target genes which in turns determines the coding of proteins and the metabolic competency of the cells. The EMSA studies revealed increased binding activity of HNF3α, HNF3β, HNF4α and HNF6 with hepatocytes cultured on Matrigel. These findings were in agreement with the results obtained by Western blotting of nuclear proteins to evidence increased level of HNF4α and HNF6.

As mentioned above, HNF4α plays a decisive role in defining metabolic competency of hepatocytes and in the control of liver regeneration. However, after isolation, the cells are severely harmed (enzyme digestion, damage to membrane bound receptors, receptor tyrosine kinases and ion channels, etc.) and therefore require proper functioning of HNF4α to restore hepatic functions [53]. Hence, a cell culture system that promotes HNF4α is highly desirable as was clearly seen for hepatocytes cultured on Matrigel. The similar regulation of the larger subunits of C/EBPα and C/EBPβ with hepatocytes cultured on either ECM is of interest. Both isoforms of C/EBPs have anti-proliferative functions and studies have shown that for proper hepatic differentiation, entry of hepatocytes in the G0 phase is essential [54]. Additionally, there are reports stating that tumor cells have reduced C/EBPα level [55]. The present study demonstrates the gene and protein expression of hepatocyte nuclear factors are not of the same dynamic processes when hepatocytes are cultured on either ECM. Apart from transcription, the level of protein is regulated by mRNA stability, post transcriptional modifications, translational efficiency, post translational modifications and stability of the protein. The effect of post transcriptional modification is also evidenced by the different expression levels of subunits of C/EBPα and C/EBPβ in the two culture systems as shown in Fig 2.

To further delineate the effects of ECM on hepatocyte differentiation, expression analysis of hepatocyte lineage markers target by TFs were performed. Hepatocyte lineage markers provide important information about the differentiation state of mesenchymal stem cells progressing towards functional hepatocyte [24]. A recent study evidenced HNF4α to function as a key regulator of epithelial-to-mesenchymal transition with repression of mesenchymal genes by HNF4α being required by hepatocyte to retain fully differentiated [56]. The hepatocyte lineage markers analyzed in the present study were HNF3β (endodermal marker), albumin, G6P (perinatal marker) and PEPCK (postnatal marker). As discussed above and with the exception of hepatocytes cultured on Matrigel for 7 days there was no significant difference in the expression levels of HNF3β both at the gene and the protein level. Similarly, albumin gene expression was not significantly different in the two culture systems. Note, the regulatory TFs for albumin include C/EBPα, HNF1α and HNF3 [48, 57] and the genes coding for G6P and PEPCK and of other enzymes of glucose metabolism were unchanged by the different ECM components. For instance, HNF3β, HNF4α, HNF6 and C/EBPβ binds to consensus sequences of the PEPCK gene to regulate its transcription [48, 49] and a recently published high resolution genome-wide scan of HNF4α recognition sites revealed >17,500 DNA binding sites with a gene/binding site ratio that differed >6-fold between chromosomes and clustered in distinct chromosomal regions amongst >6600 genes targeted by HNF4α, thus suggesting this factor to be extremely versatile [58].

Furthermore, OTC, an important enzyme of the urea cycle, was significantly up regulated on Matrigel. As expected, HNF4α that participates in OTC transcription also displayed enhanced DNA binding activity on Matrigel cultivated hepatocytes [57]. The expression of COUP-TF, a transcriptional repressor of OTC was unaffected by the different sources of ECM. The discrepancy in the expression level of TFs and their target genes may in part be due to differences in DNA binding activity of HNFs and of other regulatory proteins not studied in the present investigation. Ultimately, expression of liver-specific genes is a complex orchestrated interplay of many transcription factors, co-activators, repressor, co-repressor and epigenetic mechanisms.

Further experiments were carried out to obtain information on the metabolic competency of hepatocytes in both culture systems. Specifically, CYP enzymes play a major role in metabolism of xenobiotics and endogenous molecules such as testosterone. The Western blotting experiments revealed expression of CYP1A2, CYP12E1, CYP3A1 and CYP3A2 to be more abundant with hepatocytes cultured on Matrigel and metabolic assays with testosterone as a substrate confirmed significant higher production of 2α-hydroxytestosterone (CYP2C11), 6α-hydroxytestosterone (CYP2A1) and 7α-hydroxytestosterone (CYP2A1) with hepatocytes cultured on Matrigel while the production of 6-ß-hydroxytestosterone (catalyzed by CYP1A1, CYP2A2, CYP2C13, CYP3A1 and CYP3A2) did not differ amongst the two culture systems. In contrast, production of 2α- and 16α-hydroxytestosterone (CYP2C11, CYP2B1) was significantly increased with collagen sandwiched hepatocytes on days 5 and 8, respectively. Note, this reaction is catalyzed by CYP2C11, a major male specific CYP mono-oxygenase. Likewise, with collagen sandwiched hepatocytes the metabolism of testosterone to androstendione was initially increased but subsequently reduced and this reaction is catalyzed by 17β-hydroxysteroid dehydrogenase and 5delta-reductase to yield 5α-androstane-3,17-dione. Overall, the results suggested that cells cultured on Matrigel are more competent in metabolizing testosterone as compared to collagen cultures but differences amongst individual isoforms are also seen.

We further examined albumin synthesis over a period of 9 days and the ELISA measurements revealed similar production rates, i.e.1.9 μg/ml/2 Mio cells/24h and 2.3 μg/ml/2 Mio cells/24h for Matrigel and collagen cultured hepatocytes, respectively (data not shown). Note, the albumin mRNA expression was unchanged amongst the two culture systems.

LETFs are important regulators of the CYP gene family and the coded proteins play crucial roles in the metabolism of endogenous and foreign molecules. Iwahori et al. [59] reported that HNF4α plays a significant role in induction of CYP3A1/2 that is required for conversion of testosterone to 6α- and 6ß-hydroxytestosterone. In the present study, expression and DNA binding activity of HNF4α was significantly higher with hepatocytes cultured on Matrigel. Likewise, with Matrigel production of 6α- and 6-ß-hydroxytestosterone was significantly increased.

Moreover, the effect of Aroclor 1254 in rat liver and primary rat hepatocytes cultured on collagen and Matrigel was studied. Aroclor 1254 treatment caused a significant increase (4.6 fold) in the expression of C/EBPα in rat hepatocytes cultured on collagen. Moreover, the DNA binding activity of C/EBPα and HNF4α increased after Aroclor 1254 treatment *in vivo* as well as *in vitro* when compared to controls. In the present study, Aroclor 1254 treatment induced transcription of CYP1A1, CYP1A2 in rat liver after 3 days of a single dose. Note, the ability of Aroclor 1254 to induce transcription has been repeatedly shown [28, 60, 61]. However, differences in the doses and exposure time do not permit direct comparisons of *in vitro* and *in vivo* findings.

## Conclusion

The molecular composition of ECM influences the expression and activity of tissue specific transcription factors thereby instructing various programs of hepatocytes. The interplay of transcription factors and other regulatory proteins infer on cellular differentiation, growth and metabolism. A clear effect of ECM on the cell morphology of primary hepatocytes was observed and the discrepancies in the expression at the gene and protein level in the two culture systems are the result of transcriptional and posttranslational modifications endorsed by cellular—ECM interactions. Based on DNA binding activity and immunoblotting results particularly of HNF4α we conclude that in vitro morphology is not a good indicator of differentiation of hepatocytes. Taken collectively, the study provides evidence of the role of extracellular matrix in the selective regulation of liver specific transcription factors to influence the metabolic competency of cultured hepatocytes.

## Supporting Information

S1 Fig Expression of housekeeping genes, liver enriched transcription factors and hepatocyte-specific genes in primary rat hepatocytes cultured on Matrigel and collagen. Whisker-box plot showing average RNA intensity of various genes in primary rat hepatocytes cultured on Matrigel and collagen on day 5, 6, 7, 9 and 12. **(A)** GAPDH, 28S rRNA, **(B)** HNF-1β, HNF-3α, HNF-6, **(C)** C/EBP-γ, C/EBP-δ and CDP were significantly up-regulated in rat hepatocytes cultured on collagen sandwich as compared to Matrigel. **(D)** Ornithine transcarbamylase (OTC) expression increased significantly in cells cultured on Matrigel as compared to collagen. Depicted are 3 independent measurements taken at each time point (day 5, 6, 7, 9 and 12) and significance was determined using the student's T test. Results were considered significant at p < 0.05. M: Matrigel, C: Collagen.
(TIF)

S2 Fig DNA binding activity of liver enriched transcription factors in hepatic nuclear extracts of control animals. Depicted are EMSA assays for HNF-1α, HNF-3α, HNF-3β, HNF-3γ, HNF-4α and C/EBP-α with nuclear extracts isolated from liver of control animals. Competition assays were performed with 100-fold excess of reference (lane 2) and/or mutant probe (lane 3) for HNF4-α and C/EBP-α, respectively). Band shift assays were done with specific antibodies as indicated. C: Control, Mu: Mutant.
(TIF)

S1 Table Primer sequences and annealing temperature. (DOC)

S2 Table Primers for Aroclor 1254 experiment. (DOC)

S3 Table Antibodies used for Western blot and Gel shift assays. (DOC)

S4 Table DNA probes used for Gel shift assays. (DOC)
