# Supplementary figures and images for: Regulation of Liver Enriched Transcription Factors in Rat Hepatocytes Cultures on Collagen and EHS Sarcoma Matrices
Source: PLoS One. 2015 Apr 22;10(4):e0124867. doi: 10.1371/journal.pone.0124867 (PMC4406752; doi:10.1371/journal.pone.0124867)

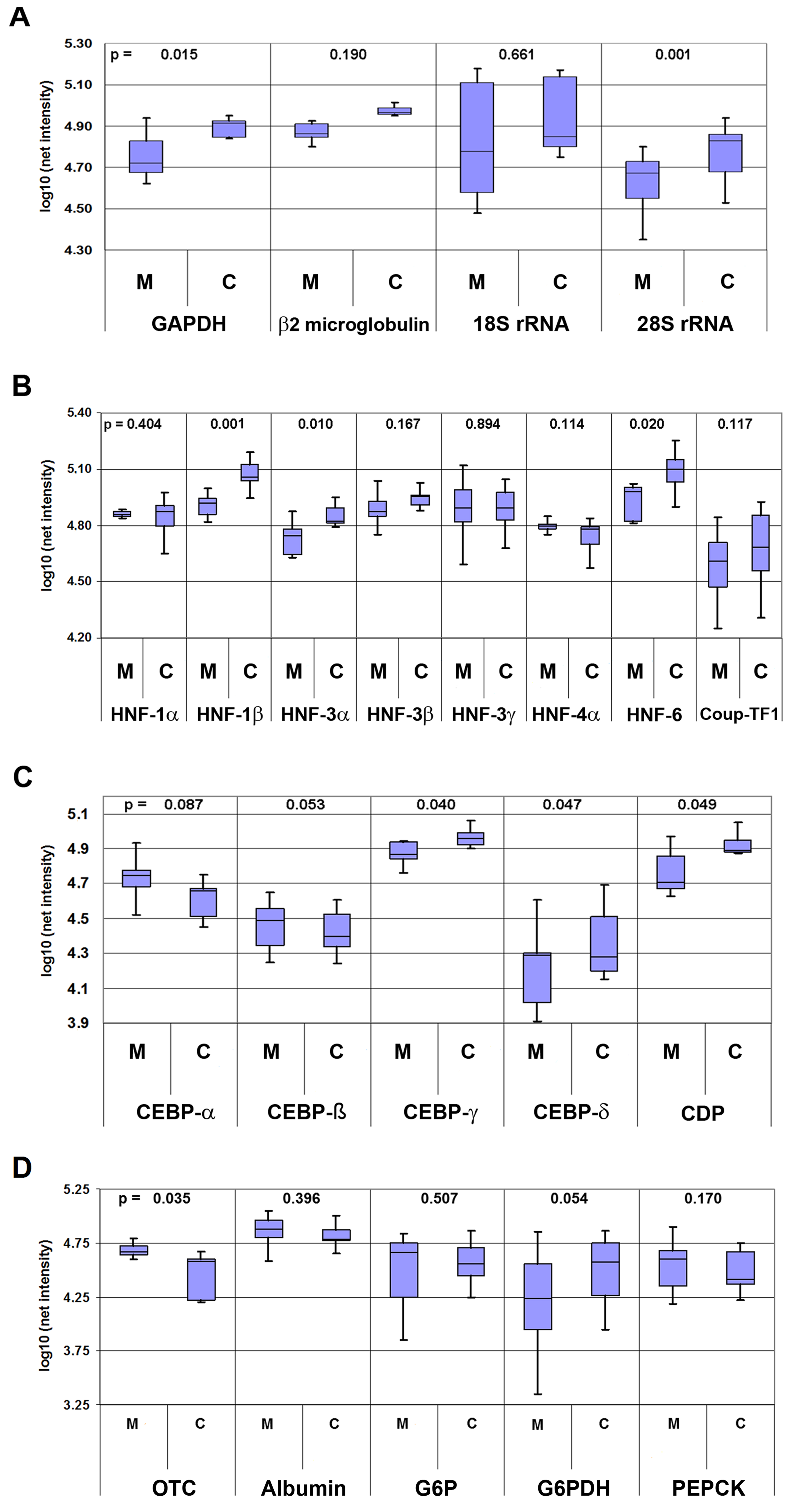

Supplement: S1 Fig — Whisker-box plot showing average RNA intensity of various genes in primary rat hepatocytes cultured on Matrigel and collagen on day 5, 6, 7, 9 and 12. (A) GAPDH, 28S rRNA, (B) HNF-1β, HNF-3α, HNF-6, (C) C/EBP-γ, C/EBP-δ and CDP were significantly up-regulated in rat hepatocytes cultured on collagen sandwich as compared to Matrigel. (D) Ornithine transcarbamylase (OTC) expression increased significantly in cells cultured on Matrigel as compared to collagen. Depicted are 3 independent measurements taken at each time point (day 5, 6, 7, 9 and 12) and significance was determined using the student's T test. Results were considered significant at p < 0.05. M: Matrigel, C: Collagen. (TIF) [file pone.0124867.s001.tif]

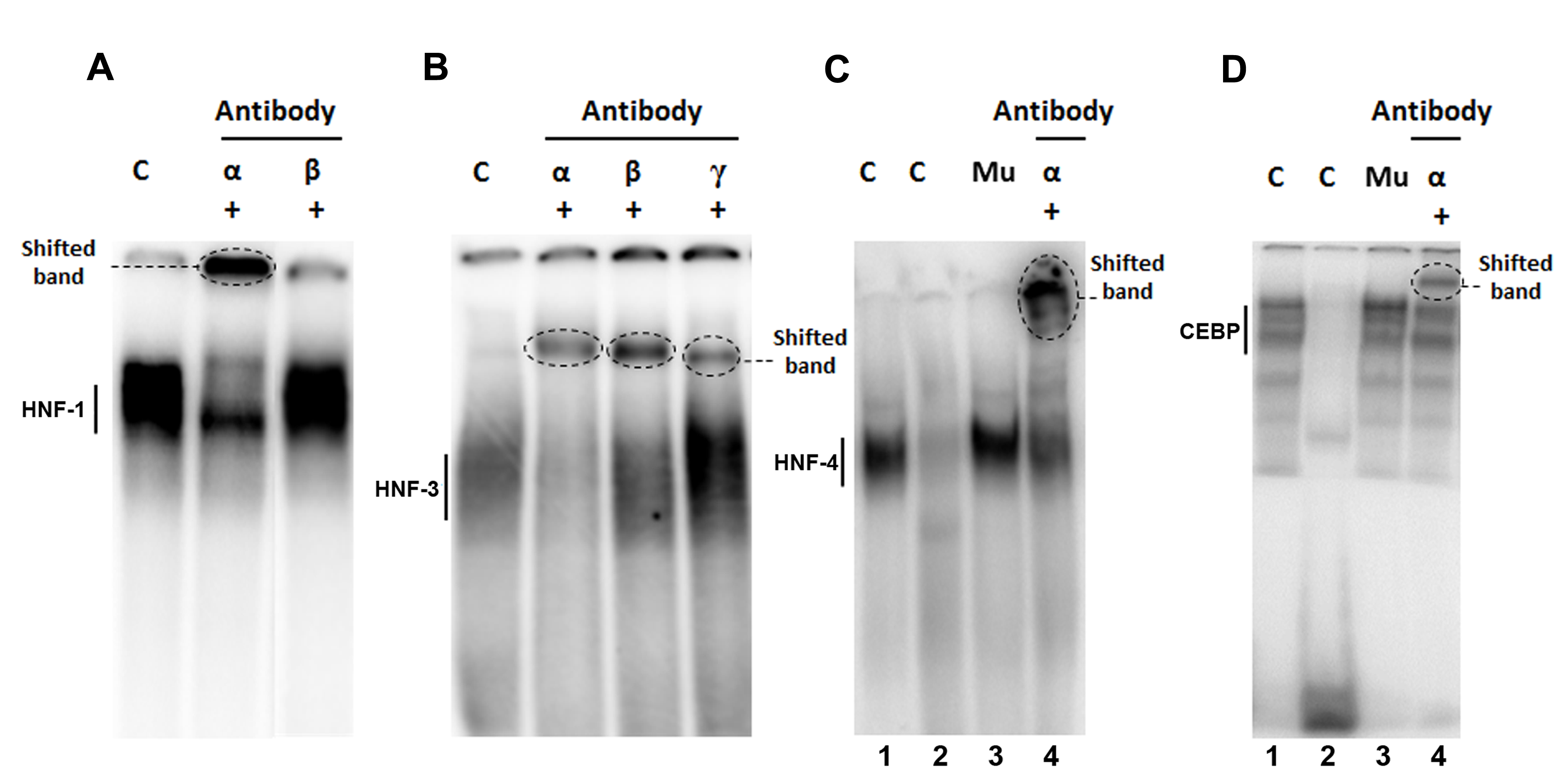

Supplement: S2 Fig — Depicted are EMSA assays for HNF-1α, HNF-3α, HNF-3β, HNF-3γ, HNF-4α and C/EBP-α with nuclear extracts isolated from liver of control animals. Competition assays were performed with 100-fold excess of reference (lane 2) and/or mutant probe (lane 3) for HNF4-α and C/EBP-α, respectively). Band shift assays were done with specific antibodies as indicated. C: Control, Mu: Mutant. (TIF) [file pone.0124867.s002.tif]
