# Supplementary material for: Regulation of Liver Enriched Transcription Factors in Rat Hepatocytes Cultures on Collagen and EHS Sarcoma Matrices
Source: PLoS One. 2015 Apr 22;10(4):e0124867. doi: 10.1371/journal.pone.0124867 (PMC4406752; doi:10.1371/journal.pone.0124867)
Supplement: S1 Table — (DOC) [file pone.0124867.s003.doc]

**S1 Table: Primer sequences and annealing temperature**

| **Gene** | **Annealing temperature** | **Fragment length** | **Primer Sequence (5’-3’)** |
| --- | --- | --- | --- |
| HNF-1α fwd | 57°C | 273 bp | TTCTAAGCTGAGCCAGCTGCAGACG |
| HNF-1α rev | 57°C | 273 bp | GCTGAGGTTCTCCGGCTCTTTCAGA |
| HNF-1β fwd | 60°C | 349 bp | CAGAGGGAGGTGGTTGATGT |
| HNF-1β rev | 60°C | 349 bp | GGTCGTAGGCCTGGTACAAA |
| HNF-3α fwd | 60°C | 303 bp | CCCTACTCCTACATCTCGCTCATC |
| HNF-3α rev | 60°C | 349 bp | CGGCTGCTTCTCACACTTGA |
| HNF-3β fwd | 57°C | 342 bp | AGCAGCAACATCATCACAGC |
| HNF-3β rev | 57°C | 342 bp | GGCCTTAAGTGTGGTGGCTA |
| HNF-3γ fwd | 57°C | 342 bp | GACTCATGCCAAACCACCTT |
| HNF-3γ rev | 57°C | 342 bp | GGATGTGGCACTGTTTCCTT |
| HNF-4α fwd | 55°C | 274 bp | GCCTGCCTCAAAGCCATCAT |
| HNF-4α rev | 55°C | 274 bp | GACCCTCCAAGCAGCATCTC |
| HNF-6 fwd | 57°C | 309 bp | AGGACTTCCCCACTATGCTCA |
| HNF-6 rev | 57°C | 309 bp | GCCACCTCTTTGGTATTGATCTC |
| COUP-TFI fwd | 60°C | 283 bp | ACAGGAACTGTCCCATCGAC |
| COUP-TFI rev | 60°C | 283 bp | ATGTTCTCGATGCCCATGAT |
| C/EBP-α fwd | 57°C | 238 bp | GCCAAGAAGTCGGTGGATAA |
| C/EBP-α rev | 57°C | 238 bp | CCTTGACCAAGGAGCTCTCA |
| C/EBP-β fwd | 57°C | 263 bp | CCTTGACCAAGGAGCTCTCA |
| C/EBP-β rev | 57°C | 263 bp | CGAAACGGAAAAGGTTCTCA |
| C/EBP-δ fwd | 60°C | 300 bp | AGTTGAGTGTGGCCTTCTCG |
| C/EBP-δ rev | 60°C | 300 bp | CAGCCATATTGTTCCGCTC |
| C/EBP-γ fwd | 60°C | 348 bp | AGGGGAAAATGGAAGGTCAT |
| C/EBP-γ rev | 60°C | 348 bp | AACAAACAAGGGGGAAAAGC |
| C/EBP-δ fwd | 60°C | 327bp | CCGACCTCTTCAACAGCAAT |
| C/EBP-δ rev | 60°C | 327bp | CTTCTGCTGCATCTCCTGGT |
| CDP fwd | 60°C | 290 bp | AACTCCAGACTCTGCAAACAGC |
| CDP rev | 60°C | 290 bp | CAACTCTACTTCTAGGCTGGATCG |
| β-2-Microglobulin fwd | 60°C | 253 bp | GTGACCGTGATCTTTCTGGTG |
| β-2-Microglobulin rev | 60°C | 253 bp | CAGTGTGAGCCAGGATGTAGAA |
| 18S rRNA fwd | 57°C | 531 bp | ACGACCAGAGCGAAAGCAT |
| 18S rRNA rev | 57°C | 531 bp | GGACATCTAAGGGCATCACAGAC |
| 28S rRNA fwd | 60°C | 320 bp | GGTGAAGAGACATGAGAGGTGTAG |
| 28S rRNA rev | 60°C | 320 bp | GAGGTTTCTGTCCTCCCTGA |
| Glc-6-P fwd | 60°C | 379 bp | AGCTCCGTGCCTCTGATAAA |
| Glc-6-P rev | 60°C | 379 bp | ATCCAAGTGCGAAACCAAAC |
| Albumin fwd | 60°C | 285 bp | CTGAACCGTCTGTGTGTGCT |
| Albumin rev | 60°C | 285 bp | GAAGTCACCCATCACCGTCT |
| GAPDH fwd | 60°C | 299 bp | GTGATGCTGGTGCTGAGTATGTC |
| GAPDH rev | 60°C | 299 bp | CAGTCTTCTGAGTGGCAGTGATG |
| OTC fwd | 60°C | 335 bp | GCAGCTACTCCAAAGGGTTATG |
| OTC rev | 60°C | 335 bp | TTCTGCCTCTGGGAACACTAAT |
| PEPCK fwd | 57°C | 359 bp | AGGAAGTGAGGAAGTTTGTGGA |
| PEPCK rev | 57°C | 359 bp | GAATGGGATGACATACATGGTG |
| Glc-6P-DH fwd | 57°C | 398 bp | TCTACCCGAAGACACCTTCATT |
