# Supplementary material for: Regulation of Liver Enriched Transcription Factors in Rat Hepatocytes Cultures on Collagen and EHS Sarcoma Matrices
Source: PLoS One. 2015 Apr 22;10(4):e0124867. doi: 10.1371/journal.pone.0124867 (PMC4406752; doi:10.1371/journal.pone.0124867)
Supplement: S2 Table — (DOC) [file pone.0124867.s004.doc]

**S2 Table: Primers for Aroclor experiment**

| **Gene** | **Forward primer** | **Reverse primer** |
| --- | --- | --- |
|  |  |  |
| CYP1A1 | CTGGTTCTGGATACCCAGCTG | CCTAGGGTTGGTTACCAGG |
| CYP1A2 | GTCACCTCAGGGAATGCTGTG | GTTGACAATCTTCTCCTGAGG |
| CYP2E1 | CTCCTCGTCATATCCATCTG | GCAGCCAATCAGAAATGTGG |
| CYP3A1 | ATCCGATATGGAGATCAC | GAAGAAGTCCTTGCTTGC |
| CYP3A2 | CGACTTGGAACCCATAGAC | GGCTTAGGGAGATTTGACATG |
| CYP4A1 | GGTGACAAAGAACTACAGC | AGAGGAGTCTTGACCTGCCAG |
| Apolipoprotein CIII | ACATGGAACAAGCCTCCAAG | ACGGCTCAAGAGTTGGTGTT |
| Cyclophillin | CTTGCCATTCCTGGACCCAA | TTTCGTGCTCTGAGCACTGG |
| Phenylalanine  hydroxylase | CTGTTGCTGGCTTACTGTCG | TACTCCTGGCAGGCTGTCTT |
