# Supplementary material for: Regulation of Liver Enriched Transcription Factors in Rat Hepatocytes Cultures on Collagen and EHS Sarcoma Matrices
Source: PLoS One. 2015 Apr 22;10(4):e0124867. doi: 10.1371/journal.pone.0124867 (PMC4406752; doi:10.1371/journal.pone.0124867)
Supplement: S3 Table — (DOC) [file pone.0124867.s005.doc]

**S3 Table: Antibodies used for Western blot and Gel shift assays**

| **Primary Antibody** | **Company** | **Article number** |
| --- | --- | --- |
|  |  |  |
| anti-HNF1α goat polyclonaI IgG | Santa Cruz | sc-6547X |
| anti-HNF3α goat polyclonaI IgG | Santa Cruz | sc-9186X |
| anti-HNF3β goat polyclonaI IgG | Santa Cruz | sc-6554X |
| anti-HNF3γ goat polyclonaI IgG | Santa Cruz | sc-5361X |
| anti-HNF4α goat polyclonaI IgG | Santa Cruz | sc-6557X |
| anti-HNF-6 goat polyclonaI IgG | Santa Cruz | sc-6559X |
| anti-CEBP-α rabbit polyclonaI IgG | Santa Cruz | sc- 61X |
| anti-CEBP-β rabbit polyclonaI IgG | Santa Cruz | sc- 150X |
| anti-CYP1A1 mouse polyclonaI IgG | Rubitec | 1A3-03 |
| anti-CYP2E1 sheep polyclonaI IgG | Chemicon International | AB-1274 |
| anti-CYP3A1 sheep polyclonaI IgG | Chemicon International | AB-1277 |
| anti-CYP3A2 sheep polyclonaI IgG | Chemicon International | AB-1275 |
| **Secondary antibody** | | |
| sheep anti-goat horseradish  peroxidase conjugated IgG | Chemicon International | AP147 P |
| goat anti-rabbit horseradish  peroxidase conjugated IgG | Chemicon International | AP132 P |
| goat anti-mouse horseradish  peroxidase conjugated IgG | Chemicon International | AP160P |
| rabbit anti-sheep horseradish  peroxidase conjugated IgG | Chemicon International | AP147P |
