# Supplementary material for: Regulation of Liver Enriched Transcription Factors in Rat Hepatocytes Cultures on Collagen and EHS Sarcoma Matrices
Source: PLoS One. 2015 Apr 22;10(4):e0124867. doi: 10.1371/journal.pone.0124867 (PMC4406752; doi:10.1371/journal.pone.0124867)
Supplement: S4 Table — (DOC) [file pone.0124867.s006.doc]

**S4 Table: DNA probes used for Gel shift assays**

| **Probe** | **Sequence (5’-3’)** | **Location** | |
| --- | --- | --- | --- |
| HNF1wtGS-fwd | ACA CGG ATA AAT ATG AAC CTT GG | HNF1 binding site -26 to -4 in HNF1-Promotor (B-site) | |
|  |  |  | |
| HNF1wtGS-rev | CCA AGG TTC ATATTT ATC CGT GT | HNF1 binding site -26 to -4 in HNF1-Promotor (B-site) | |
|  |  |  | |
| HNF3wtGS-fwd | GTT GAC TAA GTC AAT AAT CAG AAT CAG | TTR promotor -111 to -85 HNF3 S region (Transthyretin promotor) | |
|  |  |  | |
| HNF3wtGS-rev | CTG ATT CTG ATT ATT CAG TTA GTC AAC | TTR promotor -111 to -85 HNF3 S region (Transthyretin promotor) | |
|  |  |  | |
| HNF4wtGS-fwd | AAG GCT GAA GTC CAA AGT TCA GTC CCT TC | HNF4 binding site -62 to -34 in HNF1-Promotor (A-site) | |
|  |  |  | |
| HNF4wtGS-rev | GAA GGG ACT GAA CTTTGG ACT TCA GCC TT | | HNF4 binding site -62 to -34 in HNF1-Promotor (A-site) |
|  |  |  | |
| C/EBP-α GS-fwd | CTA GGG CTT GCG CAA TCT ATA TTC G |  | |
|  |  |  | |
| C/EBP-α GS-rev | C GAA TAT AGA TTG CGC AAG CCC TAG |  | |
